# Long-term Effects of Cholinesterase Inhibitors on Cognitive Decline and Mortality

**DOI:** 10.1212/WNL.0000000000011832

**Published:** 2021-04-27

**Authors:** Hong Xu, Sara Garcia-Ptacek, Linus Jönsson, Anders Wimo, Peter Nordström, Maria Eriksdotter

**Affiliations:** From the Division of Clinical Geriatrics (H.X., S.G.-P., M.E.), Division of Neurogeriatrics (L.J., A.W.), Department of Neurobiology, Care Sciences and Society, and Department of Medical Epidemiology and Biostatistics (H.X.), Karolinska Institutet; Department of Internal Medicine (S.G.-P.), Neurology Section, Södersjukhuset, Stockholm, Sweden; H. Lundbeck A/S (L.J.), Copenhagen, Denmark; Department of Community Medicine and Rehabilitation (P.N.), Geriatric Medicine, Umeå University; and Theme Aging (S.G.-P., M.A.), Karolinska University Hospital, Stockholm, Sweden.

## Abstract

**Objective:**

To investigate whether cholinesterase inhibitors (ChEIs) are associated with slower cognitive decline in Alzheimer dementia and decreased risk of severe dementia or death.

**Methods:**

Patients with Alzheimer dementia from the Swedish Dementia Registry starting on ChEIs within 3 months of the dementia diagnosis were included and compared to nontreated patients with Alzheimer dementia. In a propensity score–matched cohort, the association between ChEI use and cognitive trajectories assessed by Mini-Mental State Examination (MMSE) scores was examined with a mixed model, and severe dementia (MMSE score <10) or death as an outcome was assessed with Cox proportional hazards models.

**Results:**

The matched cohort included 11,652 ChEI users and 5,826 nonusers. During an average of 5 years of follow-up, 255 cases developed severe dementia, and 6,055 (35%) died. ChEI use was associated with higher MMSE score at each visit (0.13 MMSE points per year; 95% confidence interval [CI] 0.06–0.20). ChEI users had a 27% lower risk of death (0.73, 95% CI 0.69–0.77) compared with nonusers. Galantamine was associated with lower risk of death (0.71, 95% CI 0.65–0.76) and lower risk of severe dementia (0.69, 95% CI 0.47–1.00) and had the strongest effect on cognitive decline of all the ChEIs (0.18 MMSE points per year, 95% CI 0.07–0.28).

**Conclusions:**

ChEIs are associated with cognitive benefits that are modest but persist over time and with reduced mortality risk, which could be explained partly by their cognitive effects. Galantamine was the only ChEI demonstrating a significant reduction in the risk of developing severe dementia.

**Classification of Evidence:**

This study provides Class III evidence that for patients with Alzheimer dementia ChEIs decrease long-term cognitive decline and risk of death and that galantamine decreases the risk of severe dementia.

The acetylcholinesterase inhibitors (ChEIs) and the NMDA receptor antagonist memantine are hitherto the only specific pharmacologic treatments approved for Alzheimer dementia, the most common type of dementia.^1^ Although their benefit appears to be modest,^2,3^ a significant body of evidence supports their effectiveness for improving cognition and their cost-effectiveness.^4-12^

Degeneration of basal forebrain cholinergic neurons is one of the earliest findings in Alzheimer dementia and precedes dementia development.^13,14^ Progression of Alzheimer dementia is better correlated with cholinergic system dysfunction than amyloid plaque load.^15^ Reduction of the volume of the basal forebrain precedes changes of hippocampal volume and predicts the cortical spread of Alzheimer pathology.^16^

ChEIs work by maximizing the availability of endogenous acetylcholine in the brain.^17^ However, few randomized clinical trials (RCTs) have examined the effectiveness of ChEIs in Alzheimer dementia after 1 year of treatment^18-22^ or followed up patients beyond this point.^20^ Studies of long-term cognitive decline are difficult due to high attrition and loss to follow-up.^20^ Although not RCTs, follow-up of ChEI-treated Alzheimer dementia cohorts has shown small cognitive benefits at 2, 3, and >10 years.^23-25^ Moreover, a positive short-term response to ChEIs can delay nursing home placement.^26^ Other studies have shown associations between ChEI use and decreased risk of myocardial infarction, stroke, and death in patients with dementia.^27-30^

We conducted a longitudinal cohort study on the Swedish Dementia Registry (SveDem) to investigate whether the cognitive benefit of ChEIs in routine settings persists over the long term and whether ChEI use is associated with decreased risk of severe dementia and death.

## Methods

### Study Design and Data Source

The longitudinal cohort study includes patients with incident diagnosed dementia registered in the SveDem (svedem.se). SveDem is a web-based registry established in 2007 with the aim of registering all patients with incident dementia in Sweden and following them up annually.^12,31,32^ The baseline registration in SveDem is initiated at the time of the dementia diagnosis. The majority of patients are diagnosed and thus registered in a mild stage of dementia, but some patients do have an advanced dementia by the time they seek care and are thus diagnosed in a more advanced stage. The registry stores data on demographics, cognitive evaluation by Mini-Mental State Examination (MMSE), the type of dementia, and pharmacologic management. SveDem was merged with the National Patient Registry to include diagnoses made in specialist clinics and hospitals, the Prescribed Drug Registry, and the Total Population and Causes of Death Registry.

### Standard Protocol Approvals, Registrations, and Patient Consents 

The regional human ethics committee in Stockholm approved the study (dnr 2017/501-31). Patients are informed about registration in SveDem at the time of their dementia diagnosis. The aim of SveDem is to improve dementia treatment and care. Patients can refuse registration, obtain information on their registration any time, and withdraw consent at a later date. Any research project on SveDem data must be approved by the ethics committee. Signed consent for research, however, was not required for this study in accordance with the protocol submitted and approved by the ethics committee. Data were deidentified by Swedish authorities before delivery to the research team.

### Study Population

**Figure 1:**
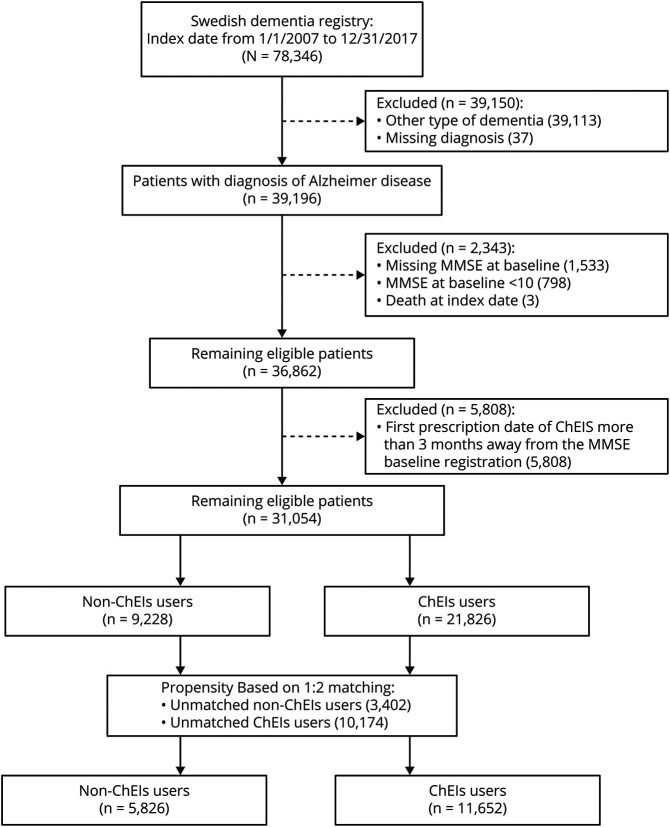
Flowchart of Included Patients ChEI = cholinesterase inhibitors; MMSE = Mini-Mental State Examination.

From 2007 to 2017, 78,346 patients with dementia were registered in SveDem; in these patients, the most common dementia types were Alzheimer dementia (31%) and mixed Alzheimer dementia (19%), followed by unspecified dementia (23%), vascular dementia (19%), Lewy body dementia (2%), frontotemporal dementia (2%), Parkinson disease with dementia (2%), and other (2%). In the present study, we included all patients with incident diagnosed Alzheimer dementia or mixed Alzheimer dementia (n = 39,196). We defined the study inclusion date as the date of the dementia diagnosis in SveDem, the date when the patient started ChEI, or the first date on which a dementia diagnosis appeared in the National Patient Registry (whichever came first). We excluded patients if data were missing on age, sex, diagnosis, or MMSE score at baseline (n = 1,533). We excluded patients with baseline MMSE score <10 (n = 789) because the indication for initiating ChEIs in Sweden is mild to moderate Alzheimer dementia (MMSE score ≥10). Finally, we excluded patients with a first prescription date of ChEI treatment >3 months from the baseline MMSE (n = 5,808) to ensure that the MMSE score was representative of the cognitive status at the start of ChEI treatment and to avoid increasing confounding by indication (figure 1). The majority (93.4%) started treatment with ChEIs on the same day of the MMSE measurement or after, while 765 (6.6%) patients were on ChEI treatment before the baseline MMSE test was performed. In Sweden, MMSE or a similar screening test is recommended when diagnosing dementia but is not required for ChEI prescription.^33^ A total 31,054 patients with Alzheimer dementia were eligible, comprising 21,826 ChEI users and 9,228 nonusers.

### ChEI Exposure

ChEI treatment (donepezil, rivastigmine, and galantamine) was defined as ChEI initiation within 3 months of the dementia diagnosis (ChEI use) (table e-1, doi.org/10.5061/dryad.2jm63xsmz). Patients who started ChEI after this initial 3-month period were excluded from analyses (figure 1). Nonusers were defined as never being treated with ChEIs during the duration of the follow-up period. In our main analysis, the exposure was assumed to be constant. This was a conservative design, attempting to limit the confounding that could occur if the patient's rate of cognitive decline influenced treatment status after initiation, imitating the intention-to-treat design of clinical trials.

We collected information on the doses of each dispensation of ChEIs over the initial 3-month period. ChEI doses were expressed as the number of defined daily doses (DDDs) present in each package or dispensation. The DDD for each drug is set by the World Health Organization and is, by definition, the “assumed average maintenance dose per day for a drug used for its main indication in adults.”^34^ To simplify comparisons, we summed the total number of DDDs dispensed during the initial 3-month period and averaged this number out over the number of days to obtain the average DDD per day. For reference, the DDD of donepezil is 7.5 mg, of rivastigmine is 9 mg (oral) or 9.5 mg (transdermal), and of galantamine is 16 mg. If multiple ChEIs were used, their DDDs were summated. For example, in clinical practice, we may start donepezil at a low dose of 5 mg; after 4 weeks, if tolerated, the dose is increased to 10 mg. For such a patient, the average DDD would be 8.3 mg after 3 months: ([5mg × 7days × 4 weeks] + [10mg × 7days × 8 weeks])/(7 days × 12 weeks).

### Covariates

Covariates were defined at the date of study entry and included age, sex, comorbid conditions (hypertension, diabetes, history of myocardial infarction, congestive heart failure, peripheral vascular disease, cerebrovascular disease, chronic pulmonary disease, chronic kidney disease, cancer, atrial fibrillation), and medications (angiotensin-converting enzyme inhibitors, angiotensin receptor blockers, β-blockers, calcium channel blocker, statins, antipsychotics, antidepressants). The definition of comorbid conditions was based on ICD-10 code.^35^ Diabetes and hypertension were in addition enriched with information on purchase of related medication up to 3 years before study entry date through the use of the Anatomical Therapeutic Chemical code A10 for antidiabetics. The ICD and Anatomical Therapeutic Chemical codes are detailed in table e-1 (doi.org/10.5061/dryad.2jm63xsmz).

### Outcomes

Baseline and follow-up MMSE scores were obtained from SveDem. Severe dementia was defined as MMSE score <10 during follow-up.^36^ The occurrence of death was obtained from the Total Population and Causes of Death Registry. Patients were followed up from study entry until the event of interest, death, or end of follow-up (October 16, 2018), whichever occurred first.

### Data Analysis

Propensity score matching was performed to balance confounders between ChEI users and nonusers. Using logistic regression models, we estimated the propensity score to receive ChEI treatment based on age, sex, baseline MMSE score, comorbid conditions, and medications. We performed 2:1 propensity score matching to pair each ChEI user to a nonuser without replacement by the nearest number matching and with a caliper of 0.01.

The cognition trajectories (MMSE score change) between ChEI users and nonusers were estimated with mixed-effects repeated-measures models of unstructured-variance-covariance matrix, which included data from all visits during the 5 years of follow-up. The model adjusted for baseline cognition, ChEI treatment (yes/no), visit time (year), and a product of ChEI treatment and visit time. Visit time had a strong nonlinear (approximately quadratic) relation with MMSE score (*p* < 0.05), so we also included a quadratic of time in the adjusted model to consider a nonlinear trend in MMSE score across time. Missing data were handled with the use of multiple imputation with chained equations in further sensitivity analysis. We in addition considered the potential effects of general attrition from those lost to follow-up due to dropout or to the presence of a competing risk before the end of follow-up such as death. We compared the cognitive trajectories between ChEI users and nonusers with 3 approaches: (1) mixed-effect model in real data; (2) multiple imputations, leveraging auxiliary variables associated with MMSE score missingness, in conjunction with mixed-effect models; and (3) inverse probability of censoring weighting in conjunction with multiple imputations and mixed-effect model. This last method was developed specifically to manage attrition in the SveDem cohort.^37^

Incidence rates per 1,000 person-years with 95% confidence intervals (CIs) were calculated for severe dementia risk (MMSE score <10) in patients who had >1 MMSE measurements and death (all patients). Cox proportional hazards regression was used to calculate hazard ratios (HRs) associated with ChEI and each outcome. In addition, we performed subgroup analyses by sex, age categories, type of dementia, and different comorbid conditions. Furthermore, we performed a competing-risk analysis for the outcomes of severe dementia with death as the competing risk. We modeled ChEI use as a continuous exposure for increasing doses in a cubic spline with each outcome. The analyses were also run separately on donepezil, rivastigmine, and galantamine.

All analyses were performed with R (r-project.org; R Foundation for Statistical Computing, Vienna, Austria) and Stata version 16.0 (StataCorp, College Station, TX).

### Data Availability

Requests for access to the SveDem data should be addressed to the registry holder and the steering committee (svedem.se).

This study provides Class III evidence that for patients with Alzheimer dementia, ChEIs decrease long-term cognitive decline and death. Galantamine in addition decreases the risk for severe dementia.

Supplementary data are available from Dryad (tables e-1–e-5 and figures e-1 and e-2): doi.org/10.5061/dryad.2jm63xsmz.

## Results

### Baseline Characteristics

Figure 1 shows how the propensity score–matched cohort was assembled. Table 1 and table e-2 show the baseline characteristics by ChEI treatment in patients with Alzheimer dementia before and after matching. Patients who were not on ChEIs were older, had lower MMSE score and more comorbid conditions such as cardiovascular disorders, and took more medications than treated patients (table e-2). Matching removed many of the significant imbalances, especially in age (standardized difference decreased from 67% to 1%), baseline MMSE score (46% to 0%), hypertension (29% to 0%), congestive heart failure (28% to 2%), history of atrial fibrillation (28% to 0%), and prescription of β-blocker (26% to 1%).

**Table 1:**
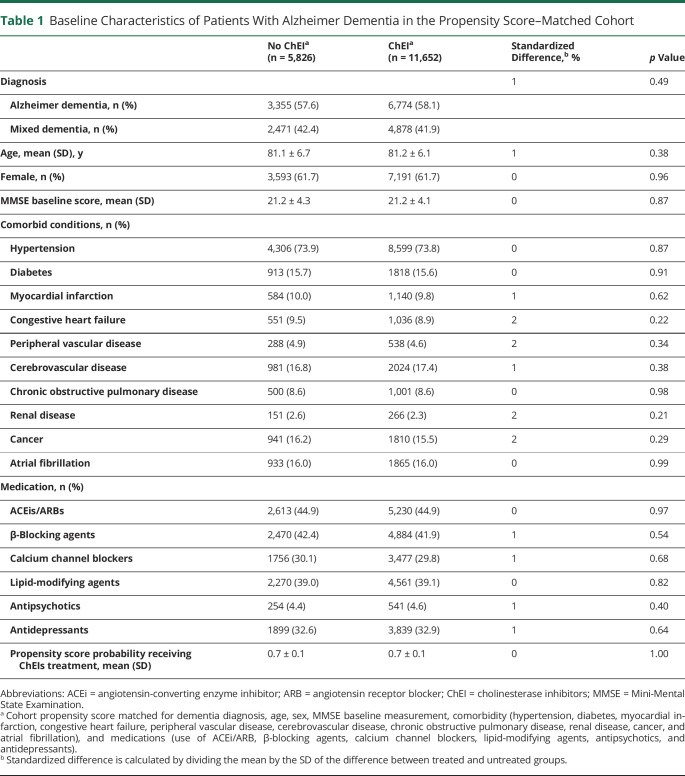
Baseline Characteristics of Patients With Alzheimer Dementia in the Propensity Score–Matched Cohort

The final propensity score–matched cohort included 11,652 ChEI users and 5,826 nonusers; 62% were women with a mean age of 81.2 ± 6.3 years. Mean baseline MMSE score was 21.2 ± 4.2 points, and the most common comorbid condition was hypertension (74%), followed by cerebrovascular disease (17%), diabetes (16%), atrial fibrillation (16%), and cancer (16%). Antihypertensive and lipid-modifying agents were highly prescribed (table 1). The median time between the dementia diagnosis and ChEI prescription was 2 (interquartile range 0–10, range 0–90) days. Among ChEI users, donepezil accounted for 62% prescriptions, followed by galantamine (21%) and rivastigmine (17%).

### ChEI Use and Long-term Cognitive Decline

In total, 27,199 measures of MMSE were available for analysis. The number of MMSE measurements for each patient was 1.6 ± 0.9 (range 1–7); 6,802 (40%) had >1 MMSE measurement taken during up to 5 years of follow-up. At baseline, the mean MMSE scores were 22.0 points in ChEI users and 21.9 points in nonusers. ChEI users presented with better MMSE scores at any visit compared to nonusers (0.13 MMSE points change slope; 95% CI 0.06–0.20 for real cohort). The average yearly reduction in MMSE score was −1.62 (95% CI −1.70 to −1.54) points for users and nonusers combined. These associations were consistent throughout several sensitivity analyses (tables 2 and 3), when estimating from raw data, and when applying a multiple imputation on missing MMSE measurement and adjusting for the inverse probability weighting for dropout during follow-up. Individual ChEI drugs showed an association with higher cognition at follow-up compared to nonusers, with galantamine presenting the largest effect size (0.18 MMSE points change, 95% CI 0.07–0.28) (table 2). There were no significant differences among different ChEIs effects on cognition (*p*_trend_ > 0.05).

**Table 2:**
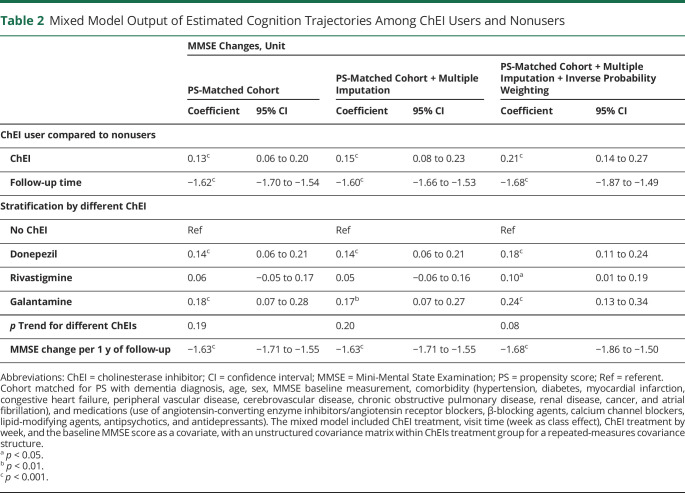
Mixed Model Output of Estimated Cognition Trajectories Among ChEI Users and Nonusers

**Table 3:**
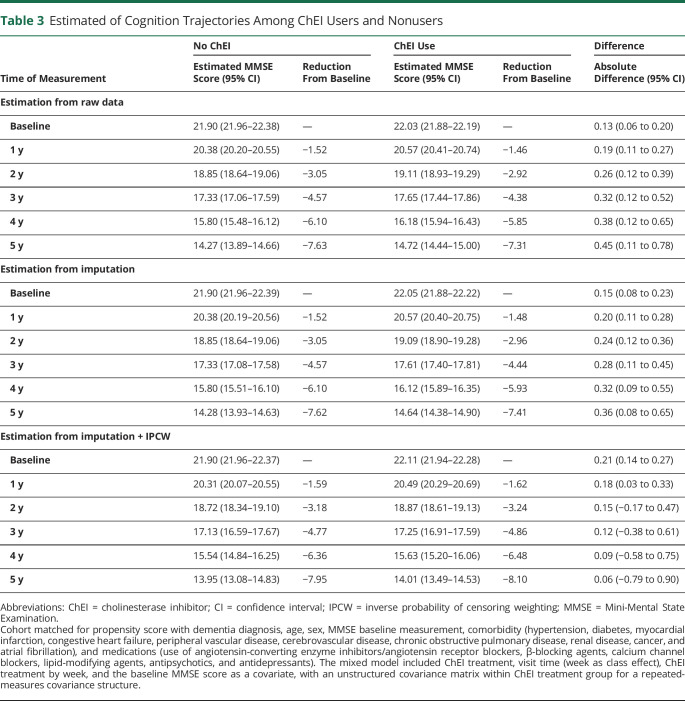
Estimated of Cognition Trajectories Among ChEI Users and Nonusers

Analyses stratified by Alzheimer dementia and mixed Alzheimer dementia diagnosis did not find any marked difference in the effects of ChEI between the Alzheimer dementia group and the mixed dementia group (0.14 [95% CI 0.05–0.24] vs 0.12 [95% CI 0.01–0.22] MMSE points change in the Alzheimer dementia and mixed Alzheimer's dementia group, respectively) (table e-3, doi.org/10.5061/dryad.2jm63xsmz). Patients who had lower cognition at the time of diagnosis (MMSE score <20) had benefits similar to those with higher MMSE scores (0.17 [95% CI 0.06–0.28] vs 0.10 [95% CI 0.01–0.18] MMSE points change in the groups with MMSE score <20 and ≥20, respectively) (table e-3).

**Figure 2:**
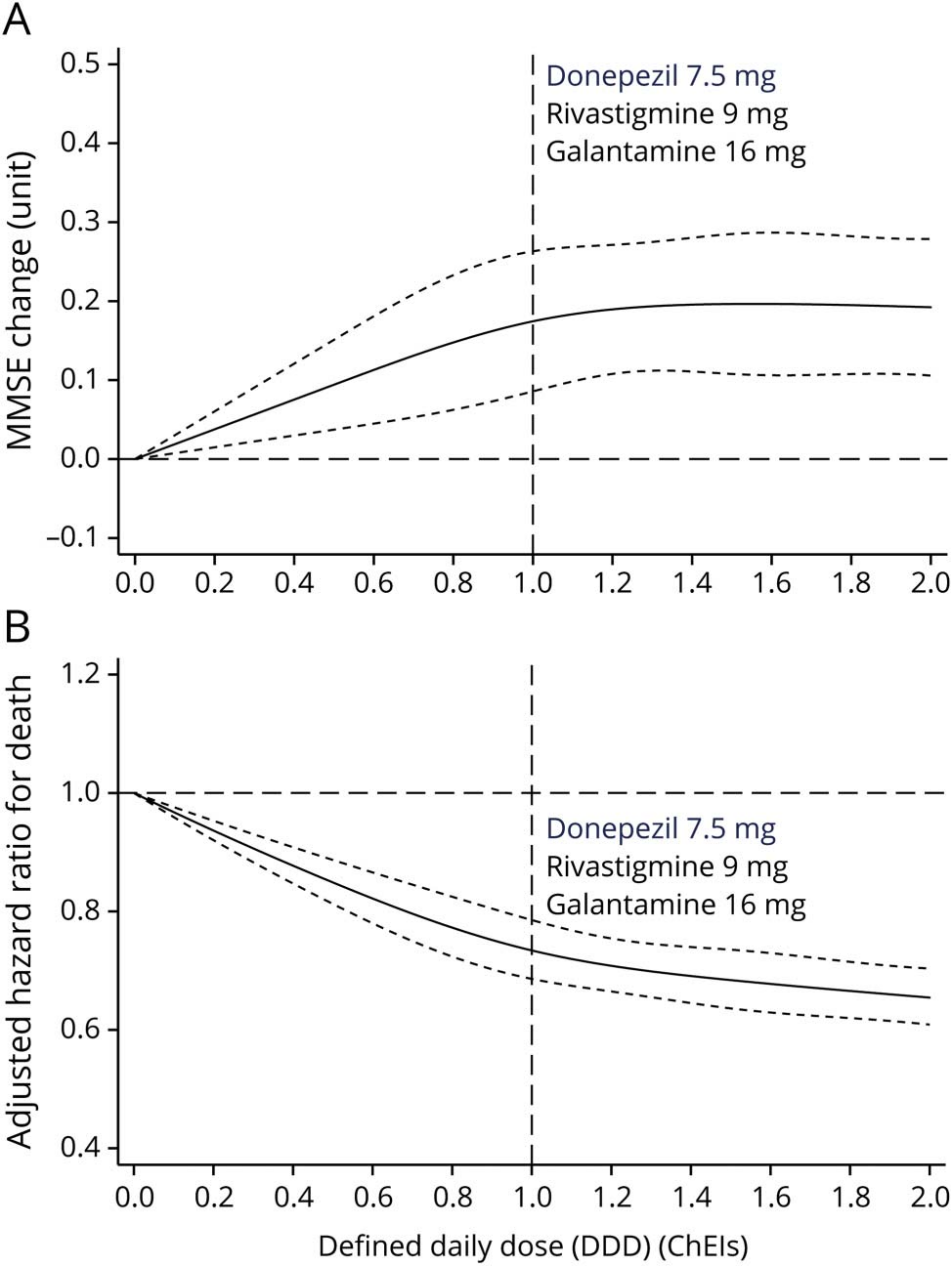
Dose Response of ChEIs Using Cubic Splines With (A) MMSE Change and (B) All-Cause Death Risk Compared With Nonuse Dose-response effect of increasing average cumulative daily dose of cholinesterase inhibitor (ChEI) compared to nonuse of ChEI: average (solid) and 95% confidence interval (dash lines). Horizontal axis represents the number of standard defined daily doses (DDDs) that patients took per day. For clarity, the DDD for each medication is shown with a vertical dotted line. For example, a patient taking galantamine 8 mg/d would be taking half the DDD of galantamine, which would be represented at the 0.5 point of the horizontal axis. In panel A, y-axis represents Mini-Mental State Examination (MMSE) score change. In panel B, y-axis represents adjusted hazard ratio for death. Model included ChEI treatment, visit time (class effect), interaction of ChEI treatment by visit, and baseline MMSE score. Reference was set at DDD 0. Cohort was matched for dementia diagnosis, age, sex, MMSE baseline score, comorbidity (hypertension, diabetes, myocardial infarction, congestive heart failure, peripheral vascular disease, cerebrovascular disease, chronic obstructive pulmonary disease, renal disease, cancer, and atrial fibrillation), and medications (angiotensin-converting enzyme inhibitors/angiotensin receptor blockers, β-blocking agents, calcium channel blockers, lipid-modifying agents, antipsychotics, and antidepressants).

Figure 2A shows the dose-response effects of ChEI on cognition. Higher dispensed doses of ChEI were associated with higher MMSE measurements during follow-up. This association was generally observed throughout the whole range of doses considered with a modest but significant effect size. When analyses on separate ChEIs were conducted, dose response of donepezil yielded results similar to the overall ChEI exposure (figure e-1A, doi.org/10.5061/dryad.2jm63xsmz). Patients taking galantamine showed improved MMSE scores at follow-up, and this was significant when dispensed doses were ≥16 mg/d (DDD ≥1). Although rivastigmine use was significantly associated with improved cognition (shown in table 2), a dose-response effect could not be demonstrated (figure e-1B and e-1C).

### ChEI and Severe Dementia Risk

When severe dementia (MMSE score <10) was considered as the outcome, there were 6,802 patients (40% of the initial population) who had at least 2 MMSE measurements. The incidence rates and the proportion of patients developing severe dementia were higher among the nonusers (incidence rate 12.2/1,000 person-years, 4.0% of all patients for nonusers vs 10.2/1,000 person-years, 3.7% of all patients for ChEI users separately). When stratified for separate ChEIs, only galantamine users had a statistically significant lower risk of severe dementia (HR 0.69, 95% CI 0.47–1.00), which was not significant for users of rivastigmine or donepezil or for ChEI users as a whole (table 3). Stratified analyses for the risk of severe dementia are presented in figure e-2A (doi.org/10.5061/dryad.2jm63xsmz). Similar associations were observed for severe dementia with the competing-risk model (table e-4).

### ChEI and Mortality

During an average of 5 years of follow-up, corresponding to 52,042 person-years, 6,055 (35%) patients died. The overall mortality rate was ≈2 times higher (115.02/1,000 person-years) compared with the age- and year-matched general Swedish population (60.59/1,000 person-years ) (table e-5, doi.org/10.5061/dryad.2jm63xsmz). Lower mortality rate was noted for ChEI users compared with nonusers (105.78/1,000 person-years vs 136.93/1,000 person-years). ChEI users had a 27% lower risk of death (HR 0.73, 95% CI 0.69–0.77) compared with nonusers. We observed significant differences among different ChEIs in regard to mortality risk (*p*_trend_ < 0.05), with an HR for galantamine of 0.71 (95% CI 0.65–0.76), for donepezil of 0.78 (95% CI 0.74–0.83), and for rivastigmine of 0.86 (95% CI 0.80–0.93) (table 4).

**Table 4:**
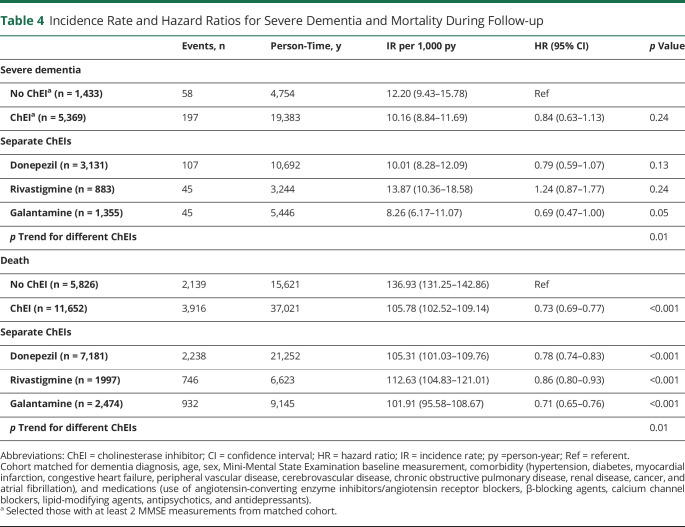
Incidence Rate and Hazard Ratios for Severe Dementia and Mortality During Follow-up

There was a graded association between dispensed average DDD of ChEIs and risk of death (figure 2B). The HRs associated with increasing average DDDs of donepezil, rivastigmine, and galantamine are graphically represented in figure e-1D through e-1F (doi.org/10.5061/dryad.2jm63xsmz). Patients who took higher dose of ChEI had lower mortality risk in a dose-dependent response. Patients taking galantamine had a lower risk of death compared with untreated patients (ratio <1) (figure e-1F).

Patients taking galantamine at any dose, >7.5 mg donepezil (figure e-1D), or >9 to 9.5 mg rivastigmine (figure e-1E) had a lower risk of death compared with untreated patients (ratio <1) (figure e-1F).

The associations between ChEI and death were consistent throughout all subgroup analyses (figure e-2B), albeit hampered in patients with peripheral vascular disease by lack of power. No interactions were observed between subgroup and ChEI in predicting mortality (all *p* > 0.05).

## Discussion

In this large longitudinal national dementia cohort, ChEI use was associated with a reduction in cognitive decline over time, and this effect was modest but persisted over the long term. ChEI use also was associated with reduced risk for mortality, which is in line with previous results from our group.^27,28^ A dose-response effect was observed for both of these outcomes. Only galantamine demonstrated a reduction in the risk for severe dementia (MMSE score <10).

Little is known about the long-term effects of ChEI on cognitive decline in Alzheimer dementia. In a 2018 Cochrane systematic review, which included 30 studies on donepezil for Alzheimer dementia, only 3 studies^18-20^ had a follow-up of 1 year, and only 1 study^18^ could be included in the meta-analysis. A recent meta-analysis (n = 16,576 patients with Alzheimer dementia; 63 RCTs) with an average duration of 8 months showed that although ChEIs had a benefit for cognition, the effect did not reach significant improvement.^38^ In the British Alzheimer's Disease 2000 study, patients with Alzheimer dementia were randomized to donepezil or placebo for 2 years. Donepezil users had 0.8 points higher MMSE scores at 2 years, but the study was underpowered and hard to interpret.^20^ In another study was performed in 5 Northern European countries, a significant advantage of donepezil treatment over placebo was observed at 52 weeks in cognition, activities of daily living, and the Progressive Deterioration Scale.^18^

The findings of our study showing significantly slower cognitive decline in patients with ChEI use are in line with results from other clinical trials. However, the magnitude of the effect appears to be somewhat smaller in our study, which is probably related to the characteristics of our cohort, the particular design, and limitations. First, we defined medication exposure within the 3 months after the dementia diagnosis, a conservative design that intended to mimic the intention-to-treat design of clinical trials and to avoid reverse causation in which the rate of cognitive decline would cause changes in medication status. Patients who were defined as treated could have stopped taking ChEIs and would still be analyzed within the treatment group, which could naturally attenuate the difference in effect. This could not be the case for nonusers; patients who were initially nonusers who started treatment after 3 months were excluded from analyses and did not contribute follow-up time in either group. In general, patients are registered in SveDem early in the disease process.^31^ In our study, patients with a baseline MMSE score >20 declined faster than patients who started with lower MMSE scores. Second, ours is a cohort study using patients from the SveDem and naturalistic follow-ups. Attrition to follow-up was high, at ≈50%. Third, we defined study inclusion at the date of dementia diagnosis or the date when the patient started ChEI (whichever came first); for this reason, a small proportion of patients (6.6% in our study) were already treated when the MMSE was performed, which would contribute to reduce the magnitude of the difference between users and nonusers.

Patients with faster cognitive decline may have higher likelihood of dropout because institutionalization, mortality, and management of the social aspects of advanced dementia probably dominate the care efforts and disrupt follow-up. Missing follow-ups would then be more frequent among severely impaired patients. This creates a situation in which patients who are followed up are more likely to start with better cognition and to decline less over time. The magnitude of the yearly decline observed in our study was −1.62 points per year, which is in line with previous clinical trials. In the National Alzheimer's Coordinating Center study, the average decline was 1.9 points in the first year and 1.5 in the second year of follow-up.^39^ In the 1-year, randomized, placebo-controlled study of patients with mild to moderate AD^18^ the placebo group declined ≈2 points per year compared to a decline of ≈0.5 points per year for the donepezil users. Meanwhile, in our study, the benefit observed with ChEIs was smaller although our conservative definition of medication exposure makes it hard to directly compare these results.

ChEIs have been proven to have symptomatic effects in Alzheimer dementia beyond those detected by standard measures of cognition. As previously shown by our group, ChEIs have been associated with reductions in myocardial infarction,^28^ stroke^27^ and mortality,^27^ while anticholinergic medications have been associated with increases in stroke and mortality risk.^40^ Better cognition may in itself be protective for mortality, but ChEIs also may have beneficial systemic effects.^27,28^ Stroke and mortality prevention in mild to moderate dementia stages is desirable, and stroke prevention could theoretically prolong independent functioning in dementia.^41-44^ Meanwhile, a recent study showed that initiation of antipsychotic treatment was reduced in those treated with ChEIs.^45^ In our study, the benefit of ChEI was similar in those diagnosed with MMSE scores <20 and those with higher MMSE scores, and the cognitive benefits persisted over time. In the Donepezil and Memantine in Moderate to Severe Alzheimer's Disease (DOMINO-AD) clinical trial, withdrawal of donepezil in patients with moderate to severe Alzheimer dementia increased the risk of nursing home placement during 12 months after treatment.^46^ Continuation was associated with better cognition.^47^ In addition, galantamine use was associated with reduced risk for severe dementia and mortality and had the largest effect size for the association with cognitive decline in our study. Galantamine is a rapidly reversible ChEI and the only ChEI that acts as an allosteric nicotinic modulator.^48,49^ This dual effect as an acetylcholinesterase inhibitor and nicotinic receptors modulator may explain its enhanced effects.

The strengths of our study are the large sample size and long cognitive follow-up. In addition, data were obtained through standard patient registration and thus reflect real-world data. We acknowledge some limitations. First, regarding the observational study design, we cannot infer causality, and we acknowledge the possibility of residual and unknown confounding. However, we controlled for the unbalanced confounders in our propensity score–matching cohort. Second, patients were considered exposed throughout the whole follow-up period according to treatment status at study entry. We attempted to mimic the intention-to-treat design of clinical trials to ensure a conservative estimate of the effects of ChEI on cognition and because of the fear of reverse causality, in which the speed of cognitive decline could influence decisions to start or withdraw treatment. Third, we acknowledge that the vascular pathology in the group with mixed Alzheimer dementia may have affected the response to ChEI on the cognition trajectories. If anything, this may have contributed to an underestimation of the effects of the ChEI presented in this study. In addition, individual patient information on reasons for prescription or side effects of ChEI and information on the dispensation form of the ChEI were not available. Fourth, the national coverage of SveDem is not absolute, and there is no clear count of how many patients develop dementia each year in Sweden. On the basis of different approximations of dementia incidence and prevalence, the coverage of SveDem for new dementia cases is estimated to be between 30% and 43%, depending on different estimations of incident cases regardless of whether they receive a diagnosis.^50^ The dementia diagnostic workup follows standard clinical practice, and few patients have a changed dementia diagnosis at follow-up,^31^ which suggests adequate diagnostic accuracy. Fifth, although the majority of patients are diagnosed early in the dementia disease process, some patients are diagnosed in a later stage, resulting in a variation of cognitive functioning at the time of the initiation of ChEI treatment. Finally, because our data were collected in real-world clinical practice, there were differences in the number of MMSE measurements performed between individuals, and these MMSE measurements were not missing at random; however, we attempted to address the potential concern of dropout by adjusting the estimates using inverse probability of censoring weighting. Results with and without adjusted weighting were similar and robust.

ChEIs are associated with cognitive benefits that are modest but persist over the long term. ChEIs are associated with reduced mortality risk, which may be partly explained by the modest cognitive effects. Galantamine was the only ChEI that demonstrated a significant reduction in the risk of developing severe dementia, in addition to presenting the strongest effect on cognition.

## Glossary

ChEI: cholinesterase inhibitor
CI: confidence interval
DDD: defined daily dose
DOMINO-AD: Donepezil and Memantine in Moderate to Severe Alzheimer's Disease
HR: hazard ratio
ICD-10: *International Classification of Diseases, 10 revision*
MMSE: Mini-Mental State Examination
RCT: randomized clinical trial
SveDem: Swedish Dementia Registry
